# Separate and Simultaneous Adjustment of Light Qualities in a Real Scene

**DOI:** 10.1177/2041669516686089

**Published:** 2017-01-01

**Authors:** Ling Xia, Sylvia C. Pont, Ingrid Heynderick

**Affiliations:** Department of Industrial Design, pi-lab (Perceptual Intelligence Lab), Delft University of Technology, The Netherlands; Department of Human Technology Interaction, Eindhoven University of Technology, The Netherlands

**Keywords:** visual light field, lighting, light direction, light diffuseness, light intensity, independent estimation, simultaneous estimation, tuning the light

## Abstract

Humans are able to estimate light field properties in a scene in that they have expectations of the objects’ appearance inside it. Previously, we probed such expectations in a real scene by asking whether a “probe object” fitted a real scene with regard to its lighting. But how well are observers able to interactively adjust the light properties on a “probe object” to its surrounding real scene? Image ambiguities can result in perceptual interactions between light properties. Such interactions formed a major problem for the “readability” of the illumination direction and diffuseness on a matte smooth spherical probe. We found that light direction and diffuseness judgments using a rough sphere as probe were slightly more accurate than when using a smooth sphere, due to the three-dimensional (3D) texture. We here extended the previous work by testing independent and simultaneous (i.e., the light field properties separated one by one or blended together) adjustments of light intensity, direction, and diffuseness using a rough probe. Independently inferred light intensities were close to the veridical values, and the simultaneously inferred light intensity interacted somewhat with the light direction and diffuseness. The independently inferred light directions showed no statistical difference with the simultaneously inferred directions. The light diffuseness inferences correlated with but contracted around medium veridical values. In summary, observers were able to adjust the basic light properties through both independent and simultaneous adjustments. The light intensity, direction, and diffuseness are well “readable” from our rough probe. Our method allows “tuning the light” (adjustment of its spatial distribution) in interfaces for lighting design or perception research.

## Introduction

Light fields in the real world are highly complex due to the spectral and spatial characteristics of light sources within a scene and the interreflections of light between surfaces, generating various shading, shadowing, and vignetting effects. Nevertheless, the human visual system is sensitive for the intensity, the primary illumination direction, and the diffuseness, which are basic properties of a light field (Khang, Koenderink, & Kappers, 2006; Koenderink, Van Doorn, Kappers, Pas, & Pont, 2003; Morgenstern, Geisler, & Murray, 2014; O’Shea, Agrawala, & Banks, 2010; Pentland, 1982; Pont & Koenderink, 2007) and concern mathematical or physical first-order properties of the light field in a spherical harmonics decomposition (Mury, Pont, & Koenderink, 2007).

Many studies into surface color and lightness perception gave indirect evidence of our awareness of the light field (Adelson, 2000; Boyaci, Maloney, & Hersh, 2003; Brainard, 1998; Brainard, Brunt, & Speigle, 1997; Coren & Komoda, 1973; Gilchrist et al., 1999; Ikeda, Shinoda, & Mizokami, 1998; Kingdom, 2011; Maloney, 2002; Robilotto & Zaidi, 2004; Schirillo, 2013; Todd, Norman, & Mingolla, 2004). Recently, several studies were done in which the awareness of the light field was tested in a more direct manner. Human observers’ awareness of the light field in a scene of an empty space was called the “visual light field” (Koenderink, Pont, van Doorn, Kappers, & Todd, 2007). We successfully probed this awareness in real scenes by introducing a real gauge object into a real scene using optical mixtures in a novel experimental setup (Ling Xia, Pont, & Heynderickx, 2013, 2014b). In that study, the task of the observers was to *judge* whether a “probe object” fitted a real scene with regard to its lighting (hereafter referred to as the fit or no-fit task). To develop the probing method and its interface further, for perception experiments and as a practical tool for lighting designers, it is necessary to know how accurately people are able to adjust the three basic properties of a light field to the extent that the “probe object” fits the real scene. Recently, research on how well participants can estimate certain properties of a light field became available. However, most of the studies focused on observers’ estimations of single properties of the illumination, with the illumination direction being studied most intensively (Koenderink & Pont, 2003; Koenderink et al., 2003; Morgenstern et al., 2014; Pentland, 1982). The independent adjustment of one lighting property is a method of controled variable tuning, and it influences appearance unilaterally. However, the light intensity, direction, and diffuseness can vary simultaneously in real environments and are confounded in appearance changes of the objects inside it. In other words, perceptually, the influences of these lighting properties on the appearance of a scene or gauge object are in no way “orthogonal,” but can interfere with each other due to basic image ambiguities. For instance, using artificial Lambertian smooth spheres and images of real rough spherical objects with various surface textures, Pont and Koenderink (2007) found that illumination direction estimates interacted with illumination diffuseness estimates, because more frontal lighting or more diffuse lighting resulted in quite similar changes in object appearance (i.e., “diffuseness-direction ambiguity”). Madsen and Donn (2006) found that this interaction formed a major “readability” problem in applying a smooth matte sphere as a light probe in architectural lighting applications. The simultaneous adjustment (and estimation) of lighting properties is thus perceptually more complicated than the independent adjustment because the tuning has more degrees of freedom while the visual effects contain ambiguities. Therefore, in this study, we investigate whether observers can estimate all first-order lighting properties simultaneously, by comparing independent (i.e., properties separated one by one) and simultaneous (i.e., properties blended together) adjustments.

In a previous study (Ling Xia et al., 2014b), the use of a rough probe (i.e., a golf ball) in comparison to a smooth sphere was found to significantly help observers detect mismatches in light direction and diffuseness between the scene and the probe. This improvement was caused by 3D texture gradients that gave information complementary to the shading (Pont & Koenderink, 2004), partly resolving the direction-diffuseness ambiguity. Light intensity discrimination was not improved by the probe roughness, as expected, since the related brightness changes did not influence the 3D texture patterns.

In this study, instead of testing whether observers can see the fit or no-fit as in our previous study, our aim was to find out whether observers can adjust the basic lighting properties in real scenes, such that the light field on a probe object fits the light field on a real scene. This is of practical interest for the design of lighting interfaces. Then, knowing that the effects of the light field properties are confounded in the appearance, the question follows whether it makes a big difference if the adjustments are done independently or simultaneously. To this aim, we performed two experiments to evaluate observers’ abilities to adjust and fit the light intensity, direction, and diffuseness using a rough probe in a real scene. In the first experiment, the three basic lighting properties were adjusted independently and in the second experiment, they were adjusted simultaneously. On the basis of former results (Koenderink, Pont, et al., 2007; Ling Xia et al., 2014b), we expect to find that both methods will work well and differ only slightly, because of possible interactions between the simultaneously inferred lighting properties. It will answer whether simultaneous adjustment of all first-order lighting properties on a rough light probe is a feasible approach in a lighting interface for lighting design and perception experiments.

## General Methods

### Experimental Setup

**Figure 1.**
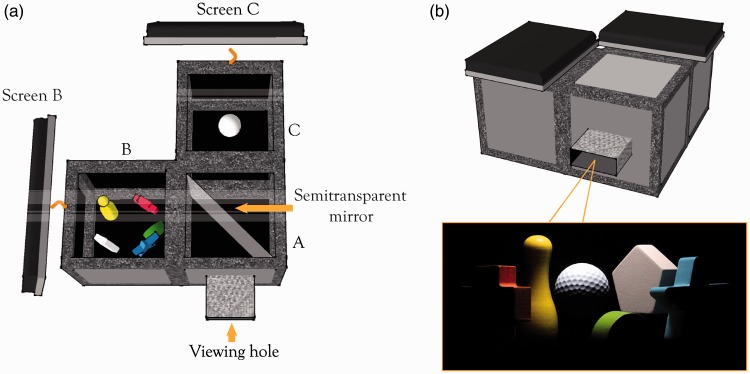
Illustration of the setup. (a) The setup consists of three 30 × 30 × 30 cm cubes, of which the inside is covered with black velvet paper (the inner width of the cubes was 25 cm excluding the width of the frames). In Cube B, we made a simple scene with five geometrical shapes. In Cube C, we placed a white-painted golf ball which served as the probe. A semitransparent mirror was placed vertically at the diagonal of box A. Two LCD screens covering the top of Cube B and Cube C provided the lighting which could thus be varied independently for the scene and the probe. (b) The setup and an optical mixture of the scene and the probe photographed through the viewing hole.

A novel type of experimental setup was used to optically introduce a real gauge object into a real scene (Figure 1(a)). The scene was located in Cube B and consisted of five colorful geometrical shapes. The probe was a white golf ball put in the center of Cube C. Because a white object has a higher albedo than an object with any other color, one of the colorful geometrical shapes was painted white to provide an anchor (Gilchrist et al., 1999). The scene and probe were optically mixed together by a semitransparent mirror put at 45° with respect to the viewing direction. When the observers looked through the viewing hole, they saw the optical mixture of the scene and probe as if they were put together (Figure 1(b)). The lighting on the scene and the probe was provided by an LCD screen on top of Cube B and Cube C, respectively (hereafter referred to Screen B and Screen C). Independent images were displayed on the two screens to provide independent lighting on the scene and on the probe. The inner width of the cubes was 25 cm, and the top of Cube B and Cube C was covered by a 930 pixels × 930 pixels square area on the LCD screens. We refer to Ling Xia et al. (2013, 2014b) for more information on the experimental setup.

### Stimuli

The intensity variation was achieved by varying the pixel value of a disk displayed on the LCD screen. The average light direction, defined by the light vector (Gershun, 1939; Mury et al., 2007) with respect to the probe, was varied by displaying a disk at different positions on the LCD screen. The light diffuseness as defined by the scale of light (Frandsen, 1989), which is derived from the comparison between the size of a light source and that of an illuminated object, was varied by changing the size of the disk on the screen. To keep the total emitted luminous flux constant for the same intensity level when changing this size, we started from the number of pixels needed to fill a disk of 6.5 cm diameter and randomly distributed these pixels over the required (larger) size. So, this created a noise pattern on the LCD image (Figure 7).

### Procedure

The experiments in this study were based on a within-subject design. In the first experiment, the observers were asked to separately adjust the light direction, diffuseness, and intensity. After a short break we started the second experiment, in which the observers were asked to simultaneously adjust these properties. The observers spent around 1 hr finishing both experiments.

The experiments were performed in a dark room. The observers looked at the optical mixture of the scene and the probe through the viewing hole. We provided different lighting stimuli on the scene. The task of the observer was to adjust the lighting on the probe to fit the scene. The observers used four arrow keys to adjust the light direction, and the up and down arrow keys to increase and decrease the light diffuseness or light intensity. Furthermore, the observers could take small steps or big steps by pressing corresponding keys on the keyboard when performing the adjustment.

Once the big step was selected, each time the key was pressed, the center of the disk was moved 30 pixels (approximately 0.8 cm) in the selected direction, or the diameter of the disk increased or decreased by 30 pixels, or the pixel value increased or decreased by 10 gray levels depending on the session. Otherwise, if the small step was used, the center of the disk was moved 10 pixels, the diameter of the disk increased or decreased by 10 pixels, or the pixel value increased or decreased by three gray levels. The big steps were selected such that the differences between the lighting effects on the probe were obvious (based on a pilot experiment with four observers) and the small steps to generate just noticeable variations of lighting effects. In Experiment 2, the participants first had to decide which lighting property (direction, diffuseness, or intensity) they wanted to vary by pressing corresponding keys. If the direction, diffuseness, or intensity exceeded a boundary, the computer gave a warning sound. The participants were able to go back and forth between the three different lighting properties, until they found the best match between the probe object and real scene.

### Participants

Six female observers and nine male observers (two of whom were the first and second author), aged between 20 and 44 years old, participated in this study. The participants were naive with respect to the setup of this experiment except for the two authors. All participants had normal or corrected-to-normal vision. They all gave written, informed consent. All experiments were done in agreement with local ethical guidelines, the Dutch Law, and the Declaration of Helsinki and were approved by the TUDelft Human Research Ethics Committee.

## Experiment 1: Adjusting Intensity, Direction, and Diffuseness Independently

We first investigated how well observers can adjust and fit the light direction, diffuseness, and intensity independently in real scenes. This experiment was divided into three sessions for the three lighting properties, and the order of these three sessions was randomized per participant. Before the session, the participant was informed on the light field property he or she had to adjust. After each session, the participant was asked to rank the five shapes inside the scene from 1 to 5 according to the information these shapes gave introspectively during the adjustment, with 1 meaning *the least information* and 5 *the most information*.

### Group I: Sensitivity for Independent Light Intensity Adjustment

#### Lighting stimuli

**Figure 2.**
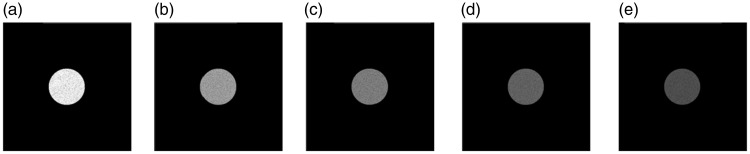
Group I stimuli: Five intensity levels on the scene were achieved by varying the gray value of a white disk with a diameter of 7 cm (264 pixels) located in the center of Screen B. (a) I1 (75.0 cd/m^2^), (b) I2 (60.0 cd/m^2^), (c) I3 (47.4 cd/m^2^), (d) I4 (36.7 cd/m^2^), and (e) I5 (28.9 cd/m^2^).

The stimuli in Group I were designed to investigate how sensitive observers were to variations in light intensity. A disk with diameter D1 (7 cm and 264 pixels) and varying gray value was displayed in the center of Screen B to serve as a light source with different intensity level on the scene. Five levels of intensity were used, marked from I1 to I5 (with I1 > I2 > I3 > I4 > I5, see Figure 2). Because of the nonlinear response of the human visual system to luminance, we adopted a constant difference in the number of Just Noticible Difference (JNDs) between the adjacent luminance levels, namely, 30 JNDs (Barten, 1992; Xu, Saha, Guarnieri, Ramponi, & Badano, 2008). The pixel values, the luminance values, and the JND values for each of the intensity levels are listed in Table 1. It should be noted that the different light intensity levels were generated by changing the pixel values on the screen, which were parameterized by luminance values of the sources. Although different terms are used here, they result in the same brightness changes on the object’s or scene’s appearance (light follows the superposition principle, so if the luminance of the source is increased by a certain factor, the luminances in the scene also increase by that factor).

**Table 1.** The Five Intensity Levels of the Light Source on the Scene That Were Used in Stimuli Group I.

Intensity levels	I1	I2	I3	I4	I5
Pixel value	255	169	135	106	85
Luminance (cd/m^2^)	75.0	60.0	47.4	36.7	28.9
JND	441	411	381	351	321

*Note.* The pixel values, luminance values, and their JND values are listed.

On the probe side, a disk with the same diameter was displayed in the center of Screen C, whose luminance value was randomly generated between 0 and 89 cd/m^2^. The task of the observers was to adjust the intensity of the light source on the probe to fit the scene.

Each stimulus was repeated for three times, resulting in 5 × 3 = 15 trials for each participant. Since we weren’t interested in differences between participants (and for intensity, diffuseness, and tilt angle, we found no statistically significant differences between observers, so we nowhere discuss this except shortly in the “Discussion” section) and since we didn’t distinguish inter- from intra-variance in participants, we considered all measurements independently in the statistical analyses. The same applies to the data analysis for all the repeated stimuli in this study.

#### Results

**Figure 3.**
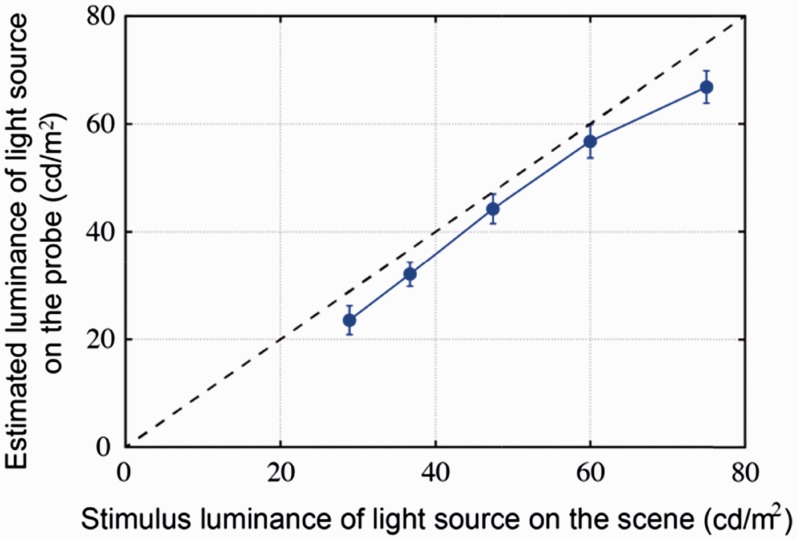
Mean of the adjusted luminance on the probe as a function of the luminance on the scene. The error bars show the 95% confidence interval (for *N* = 45 measurements).

Table 2 shows the luminance values of the light source on the scene and the mean of the corresponding adjusted luminance values on the probe. Their relationship is illustrated in Figure 3. We noticed that the adjusted luminance on the probe was well in line with the luminance on the scene except for a slight offset.

**Table 2.** The Luminance for the Scene and the Average Luminance for the Probe Adjusted by the Observers.

Luminance (cd/m^2^)	I1	I2	I3	I4	I5
Scene	75.0	60.0	47.4	36.7	28.9
Probe	66.8	56.8	44.3	32.1	23.6
*SD*	10.0	10.4	9.1	7.5	8.9

A repeated-measures analysis of variance (ANOVA) showed that the fitted intensity on the probe was significantly affected by the intensity level on the scene (*F*(4, 176) = 242.83, *p* < .001). Besides that, the posthoc test revealed that the fitted intensity for each intensity level from I1 to I5 was statistically significantly different from each other. A simple linear regression resulted in an R^2^ of 0.74 with a systematic offset of –2.5 cd/m^2^. Thus, observers could quite accurately distinguish all intensity levels we used as stimulus on the scene.

### Group II: Sensitivity for Independent Light Direction Adjustment

#### Lighting stimuli

**Figure 4.**
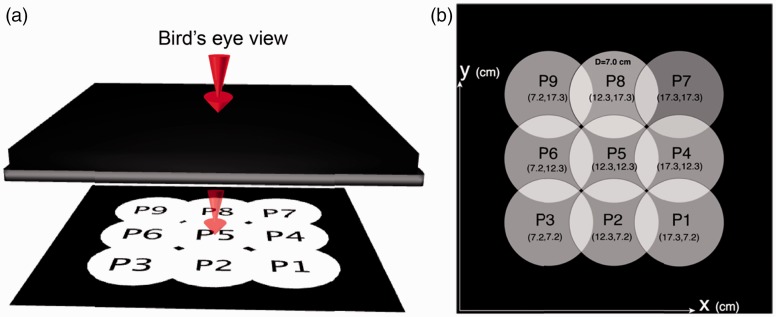
(a) Illustration of the bird’s eye view of Group II stimuli on the scene: Variation in direction was achieved by changing the position of a displayed white disk. Nine positions from P1 to P9 were used, as shown in the figure. The diameter of the disk was 264 pixels, and the width of the full window was 930 pixels (25 cm). (b) Detailed information of the disk’s positions.

The stimuli in Group II were designed to investigate how sensitive observers were to variations in light direction. A disk was displayed in one of nine different positions on Screen B. In this specific setup, the position of the disk is a convenient parameterization of the light direction with respect to the probe. The bird’s eye view of these nine positions is depicted in Figure 4, including their labels from P1 to P9 and their x and y coordinates on the screen. The positions of the disk were selected within a certain distance from the edges of the cube to make sure that there was enough space to adjust the setting of the light direction. On the probe side, a disk was displayed in a random position on Screen C. The task of the observer was to adjust the position of the light source on the probe to fit the scene.

Each stimulus was repeated for three times, resulting in 9 × 3 = 27 trials for each participant.

#### Results

**Figure 5.**
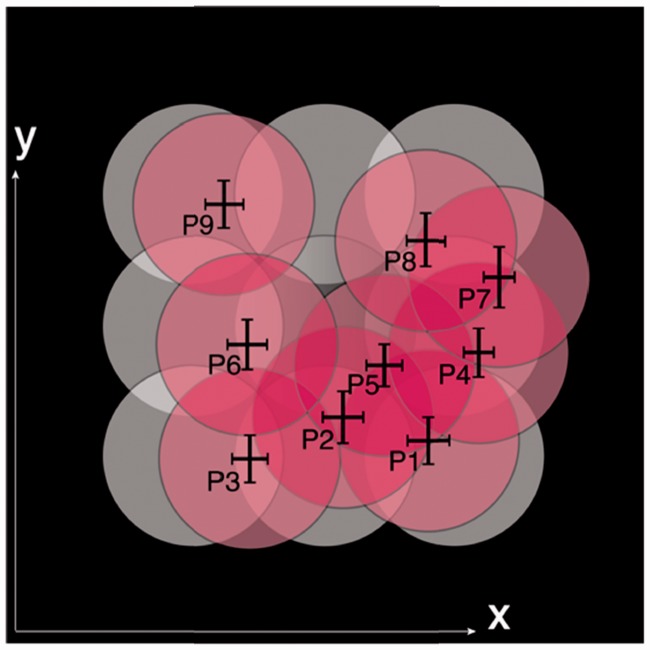
The white disks represent the positions of the light source above the scene, and the pink disks represent the averaged positions of the light source above the probe, inferred by the observers. The error bars on the pink disks show the 95% confidence intervals (for *N* = 45 measurements).

Figure 5 illustrates the positions of the light source above the scene (white disks) and the estimated positions of the light source above the probe (pink disks). The estimated positions were averaged across all 15 observers. The error bars on the pink disks show the 95% confidence intervals.

**Figure 6.**
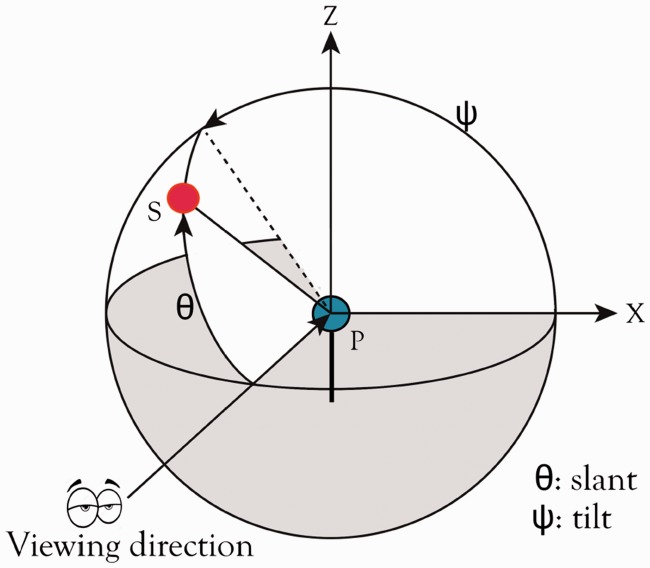
Illustration of slant and tilt. The red spot represents the light source and the blue spot the probe. The slant θ is defined as the angle between the viewing direction and the vector from the center of the probe to the center of the light source (PS). The tilt ψ is defined as the angle between the positive x-axis and the projection of PS on the surface XPZ.

The position of the light source was converted into its direction with respect to the probe, which is defined by two angles, namely, the slant and tilt, as described in Figure 6. The slant of the light source is the angle between the viewing direction and the vector from the center of the probe to the center of the light source (PS). The tilt of the light source is the angle between the positive x-axis and the projection of PS on the surface XPZ.

Both the slant and tilt angles for the nine light sources above the scene and for the inferred positions above the probe were calculated, as listed in Tables 3 and 4, respectively. The slant of the light source on the scene was approximately 74° for the front row (P1, P2, and P3 in Figure 4), 90° for the second row (P4, P5, and P6), and 106° for the back row (P7, P8, and P9). Comparing these values to the inferred slants, we found the biggest difference for P7, namely, 10° with a standard deviation of 12°. Furthermore, there is a significant difference between observers for the estimated slant angles (*F* = 3.931, *p* < .001), with four observers evaluating the slant angles lower than others and one observer evaluating the slant angles higher than others. Similarly, the tilt of the light source was 73° for the right column (P1, P4, and P7 in Figure 4), 90° for the middle column (P2, P5, and P8), and 107° for the left column (P3, P6, and P9). We compared these values to the fitted tilts on the probe and found that the biggest difference was 13° for P8, with a standard deviation of 8°. Furthermore, we found that the standard deviations of the inferred slant angles were slightly larger than those of the inferred tilt angles. The results indicated that, generally, the observers were able to estimate the light direction in our scenes.

**Table 3.** The Slant Angle of the Nine Light Sources on the Scene and the Corresponding Mean Angle on the Probe as Inferred by the Participants.

Slant	P1	P2	P3	P4	P5	P6	P7	P8	P9
Scene	74°	73°	74°	90°	90°	90°	106°	107°	106°
Probe	76°	78°	73°	87°	85°	88°	96°	100°	105°
*SD*	9.8°	11.3°	9.9°	10.3°	9.2°	10.7°	12.4°	11.2°	9.8°

**Table 4.** The Tilt Angle of the Nine Light Sources on the Scene and the Corresponding Mean Angle on the Probe as Inferred by the Participants.

Tilt	P1	P2	P3	P4	P5	P6	P7	P8	P9
Scene	73°	90°	107°	73°	90°	107°	73°	90°	107°
Probe	76°	87°	100°	70°	82°	100°	68°	77°	103°
*SD*	9.1°	9.2°	7.9°	6.3°	8.1°	8.7°	6.5°	8.4°	8.2°

We performed a 3 (stimulus row) × 3 (stimulus column) repeated measures ANOVA for the inferred slants and tilts, respectively. For the inferred slants, we found a significant main effect of the stimulus row (*F*(2, 88) = 166.95, *p* < .001). The posthoc test revealed that the inferred slants in the front row were significantly smaller than those in the middle row (*p* < .001), and the inferred slants in the middle row were significantly smaller than those in the back row (*p* < .001). For the inferred tilts, we found a significant main effect of the stimulus column (*F*(2, 88) = 439.74, *p* < .001). The posthoc test showed that the tilts for the right column were significantly smaller than those in the middle column (*p* < .001), and the tilts for the middle column were significantly smaller than that in the left column (*p* < .001). These results indicate that generally, the observers were able to make a distinction between the slants in different stimulus rows and the tilts in different stimulus columns.

Surprisingly, we also found a significant effect of the stimulus row on the tilt estimation (*F*(2, 88) = 17.83, *p* < .001). The posthoc test revealed that the fitted tilts of the front row were significantly larger than those of the middle row (*p* = .001) and the back row (*p* < .001). Both for the estimation of slant and tilt, a significant interaction between the stimulus row and the stimulus column was found (slant: *F*(4, 176) = 7.36, *p* < .001; tilt: *F*(4, 176) = 10.66, *p* < .001). This interaction together with the influence of the stimulus row on the tilt estimation were consistent with the observation in Figure 5 that the inferred position of the light source on the probe was systematically contracted to the back right corner.

The observers ranked the five shapes inside the scene according to the information they thought these shapes gave (Ling Xia and Sylvia C. Pont did not answer the questionnaire). The bowling pin was ranked 10 times out of the 13 answers as the shape providing the most information for the light direction estimation. This introspective result seems to agree with the measurements; the estimated direction of the light source on the scene was found to be contracted to the back right corner where a white pentagon was located, but not near the bowling pin. In another study, scene layout and content were shown to influence the inferred light direction in real scenes (Ling Xia, Pont, & Heynderickx, 2014a, 2016a).

### Group III: Sensitivity for Independent Light Diffuseness Adjustment

#### Lighting stimuli

**Figure 7.**
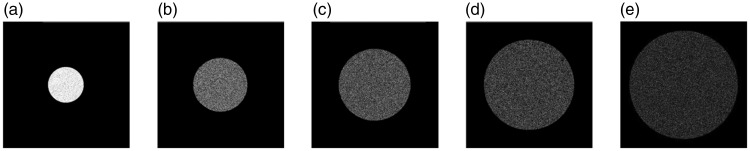
Group III stimuli on the scene: Five diffuseness levels were achieved by changing the size of a white disk on Screen B, while keeping the total number of white pixels constant. (a) D1 (7 cm), (b) D2 (10.5 cm), (c) D3 (14.0 cm), (d) D4 (17.6 cm), and (e) D5 (21.1 cm).

The stimuli in Group III were designed to investigate how sensitive the observers were to variations in light diffuseness. To this end, a disk with a diameter of 264 pixels (7.0 cm), 398 pixels (10.5 cm), 532 pixels (14.0 cm), 666 pixels (17.6 cm), or 800 pixels (21.1 cm; a stepwise increase of 134 pixels) was displayed in the center of Screen B (Figure 7). The five diffuseness levels were labeled from D1 to D5 and characterized with the scale of light. Frandsen et al. proposed the scale of light as a diffuseness measure derived from the comparison between the size of a light source and that of an illuminated object. The scale of light is quantified using the ratio of the area of the semishadow on a sphere (i.e., area receiving a varying amount of light from the source) to the area of the whole sphere (Frandsen, 1989; L. Xia, Pont, & Heynderickx, 2016b). The resulting scale of light value for the diffuseness levels from D1 to D5 are listed in Table 5.

**Table 5.** The Diffuseness Levels on the Scene Used in Stimuli Group III, and Their Scale of Light Values, Calculated According to the Definition by Frandsen, 1989.

Diffuseness levels	D1	D2	D3	D4	D5
Diameter (pixel)	264	398	532	666	800
Diameter (cm)	7.0	10.5	14.0	17.6	21.1
Scale of light (%)	21	30	39	47	54

On the probe side, a white disk with a randomly generated diameter between 250 pixels and 930 pixels was displayed in the center of Screen C. The task of the observer was to adjust the diffuseness on the probe to fit the scene. Although the diameter of the light sources varied, the total number of white pixels inside each light source was kept constant to make sure they had the same total emitted luminous flux.

Each stimulus was repeated for three times, resulting in 5 × 3 = 15 trials for each participant.

#### Results

**Figure 8.**
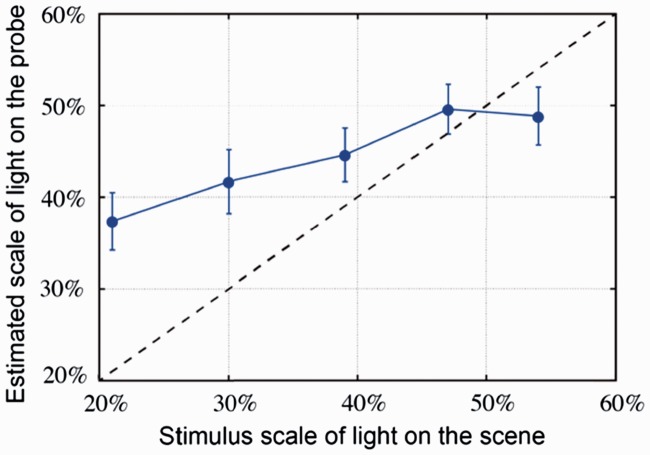
Mean of the fitted diffuseness levels (parameterized by the scale of light) on the probe as a function of the diffuseness levels on the scene. The error bars show the 95% confidence interval (for *N* = 45 measurements).

Table 6 gives the comparison between the diffuseness levels (parameterized by the scale of light) on the scene and the corresponding adjusted values on the probe averaged across the observers. The relationship is illustrated in Figure 8. Overall, the adjusted diffuseness on the probe increased as the diffuseness levels on the scene increased. However, the adjusted diffuseness levels on the probe were generally larger than the diffuseness levels on the scene and contract toward the range 37% to 50%.

**Table 6.** The Veridical Diffuseness Levels on the Scene (Parameterized by the Scale of Light) and the Mean Diffuseness Levels on the Probe as Inferred From the Participants.

Scale of light	D1 (%)	D2 (%)	D3 (%)	D4 (%)	D5 (%)
Scene	21	30	39	47	54
Probe	37	42	45	50	49
*SD*	10	12	10	9	11

We performed a repeated measures ANOVA to test the differences between adjusted diffuseness levels. We found that the adjusted diffuseness levels were significantly affected by the diffuseness levels on the scene (*F*(4, 176) = 11.14, *p* < .001). The posthoc test revealed that the inferred D1 was not significantly different from the inferred D2, the inferred D2 was not significantly different from the inferred D3, and the inferred D3 was not different from the inferred D4 and D5. This indicates that the observers could not detect all the diffuseness levels we simulated. A simple linear regression yielded a low R^2^ of 0.157. Participants’ predicted diffuseness levels increased slower than the stimuli’s diffuseness with a slope of 0.38 and a systematic offset of 0.30.

## Experiment 2: Adjusting Direction, Diffuseness, and Intensity Simultaneously

The aim of Experiment 2 is to investigate whether the observers can adjust the light direction, diffuseness, and intensity on a rough probe simultaneously to fit a real scene. Adjusting the three lighting properties simultaneously might seem challenging because it requires the ability to discriminate the lighting effects for each of these three properties.

### Lighting Stimuli

**Figure 9.**
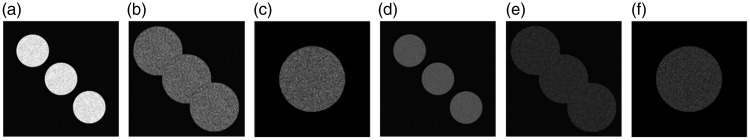
Group IV stimuli: three positions (P1, P5, P9), two diffuseness levels (D1 and D2) and one extra diffuseness level (D3) on P5, and two intensity levels (I1 and I4), resulting in 14 different stimuli in total. (a) D1 with I1 in P1, P5, and P9, (b) D2 with I1 in P1, P5, and P9, (c) D3 with I1 in P5, (d) D1 with I2 in P1, P5, and P9, (e) D2 with I2 in P1, P5, and P9, and (f) D3 with I2 in P5.

The stimuli in Group IV are shown in Figure 9. The disk was put in one of three positions: P1 (the front right corner; slant: 74°; tilt: 73°), P5 (in the center; slant: 90°; tilt: 90°), or P9 (the back left corner; slant: 106°; tilt: 107°). In each of the three positions, the disk varied between two diffuseness levels: D1 (diameter: 7 cm; scale of light of 21%) and D2 (diameter: 10.5 cm; scale of light of 30%). For the disk in the center (P5), one more diffuseness D3 (diameter: 14.0; scale of light of 39%) was added. Two intensity levels were used: I1 (75.0 cd/m^2^) and I4 (36.7 cd/m^2^). Thus, there were 2 × 3 × 2 + 1 × 2 = 14 stimuli on the scene in total.

On the probe side, a disk with random diameter between 250 and 600 pixels and random luminance between 0 and 89 cd/m^2^ was displayed in a random position. The task of the observers was to adjust the direction, diffuseness, and intensity on the probe simultaneously to make it fit the scene.

Each stimulus was repeated for three times, resulting in 14 × 3 = 42 trials for each participant.

### Results

The results for the fitted slant, tilt, diffuseness (parameterized by the scale of light), and intensity (parameterized by luminance) for the light source on the probe are listed in Table 7. The average fitted slants and tilts were all within 9° of the veridical angles. The standard deviation was within 11°, and the standard deviation for fitted slants was bigger than that for fitted tilts. Generally, the fitted diffuseness on the probe increased as the diffuseness on the scene increased, and the fitted intensity on the probe increased as the intensity on the scene increased.

**Table 7.** The Mean-fitted Slant, Tilt, Diffuseness, and Intensity Levels of the Light Source on the Probe.

Direction (slant and tilt)	Diffuseness (scale)	Intensity (cd/m^2^)	*N*	Slant		Tilt		Diffuseness (scale)		Luminance (cd/m^2^)	
				Fitted	*SD*	Fitted	*SD*	Fitted	*SD*	Fitted	*SD*
P1(74°,73°)	D1(21%)	I4(36.7)	45	81.2°	11.2°	78.8°	6.9°	31%	8.4%	22.9	13.8
**P1(74°,73°)**	**D1(21%)**	**I1(75.0)**	**45**	**79.4°**	**9.5°**	**75.3°**	**7.2°**	**29%**	**8.4%**	**68.9**	**14.1**
P1(74°,73°)	D2(30%)	I4(36.7)	45	79.5°	9.2°	79.1°	6.8°	32%	10.0%	30.0	14.4
P1(74°,73°)	D2(30%)	I1(75.0)	45	78.4°	8.6°	76.7°	5.9°	31%	7.2%	67.0	13.6
**P5(90°,90°)**	**D1(21%)**	**I4(36.7)**	**45**	**85.0°**	**8.6°**	**81.4°**	**7.3°**	**32%**	**8.6%**	**40.4**	**12.7**
**P5(90°,90°)**	**D1(21%)**	**I1(75.0)**	**45**	**87.1°**	**6.8°**	**81.7°**	**7.0°**	**32%**	**9.5%**	**71.7**	**11.4**
P5(90°,90°)	D2(30%)	I4(36.7)	45	88.8°	9.3°	84.1°	8.7°	34%	10.2%	38.4	11.6
**P5(90°,90°)**	**D2(30%)**	**I1(75.0)**	** 45**	** 88.1°**	** 7.8°**	** 82.1°**	** 6.6°**	** 34%**	** 8.9%**	** 66.1**	** 12.3**
P5(90°,90°)	D3(39%)	I4(36.7)	45	87.0°	10.4°	84.9°	7.5°	35%	10.4%	37.8	18.6
**P5(90°,90°)**	**D3(39%)**	**I1(75.0)**	** 45**	** 85.3°**	** 7.2°**	** 83.7°**	** 6.0°**	** 38%**	** 10.4%**	** 63.2**	** 13.2**
P9(106°,107°)	D1(21%)	I4(36.7)	45	100.8°	11.0°	101.1°	6.6°	30%	7.9%	33.7	10.8
**P9(106°,107°)**	**D1(21%)**	**I1(75.0)**	** 45**	** 101.9°**	** 8.7°**	** 101.9°**	** 7.2°**	** 28%**	** 8.7%**	** 57.0**	** 14.9**
P9(106°,107°)	D2(30%)	I4(36.7)	45	98.2°	9.8°	99.4°	7.0°	32%	10.1%	32.8	12.5
P9(106°,107°)	D2(30%)	I1(75.0)	45	99.3°	9.7°	101.9°	6.6°	30%	8.1%	51.1	18.7

*Note.* The rows in bold indicate stimuli that were used both in Experiments 1 and 2. Results for these stimuli will be compared at the end of this article (“Discussion” section).

Since the light source in position P5 had one more diffuseness level D3, we divided the statistical analysis into two parts: In Part 1, we analyzed the influence of all three properties (three positions: P1, P5, and P9; two diffuseness levels: D1 and D2; two intensity levels: I1 and I4) on the adjustments. In Part 2, we analyzed the influence of three diffuseness levels (D1, D2, and D3) and two intensity levels (I1 and I4) of the source in P5.

#### Part 1

**Figure 10.**
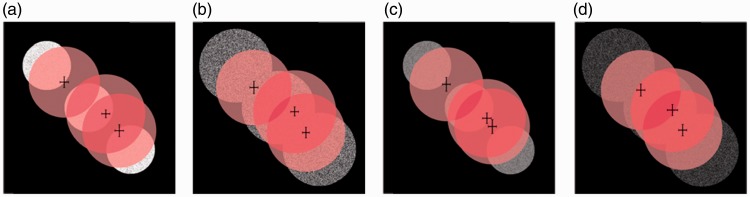
The white disks represent the light source on the scene, and the pink disks represent the mean light source on the probe estimated by the observers. Light sources in position P1, P5, and P9 with (a) diameter D1 and intensity level I1, (b) diameter D2 and intensity level I1, (c) diameter D1 and intensity level I4, and (d) diameter D2 and intensity level I4. The error bars on the pink disks show the 95% confidence intervals (for *N* = 45 measurements).

From the illustrations in Figure 10 we noticed that, in general, the fitted directions of the light source on the probe (pink disks) aligned with the light source direction on the scene (white disks). The fitted directions tended to contract toward the center of the screen. In Figure 10 (a) and (c), the fitted diameters of the light source on the probe were much larger than the diameter D1 of the light source on the scene.

**Figure 11.**
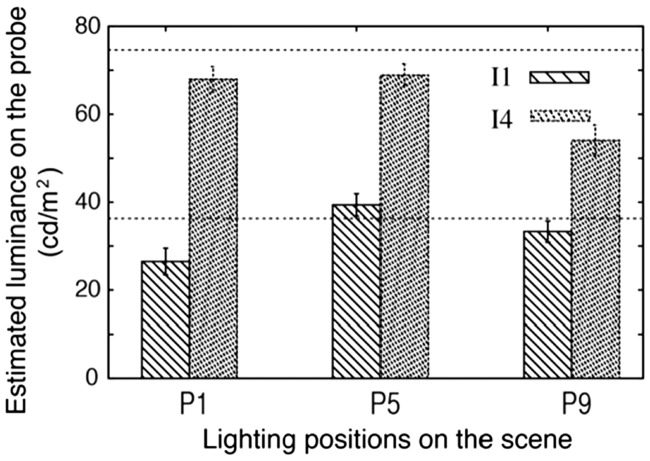
Mean-fitted luminance on the probe for I4 (the most densely dashed bars) and I1 on the scene in position P1, P5, and P9 averaged over two diffuseness levels (D1 and D2). The error bars show the 95% confidence intervals (for *N* = 90 measurements). The dashed horizontal lines show the veridical values of I1 and I4.

Figure 11 shows the fitted luminance on the probe for I1 and I4 in positions P1, P5, and P9, averaged over two diffuseness levels (D1 and D2). The veridical luminance values of I1 and I4 are marked with dashed lines. The fitted luminance values correlate well with the veridical values and again show (in most cases) a negative offset as in Experiment 1.

We performed a 3 (position) × 2 (diffuseness) × 2 (intensity) repeated measures ANOVA for the fitted slant, tilt, diffuseness, and intensity. We found a significant main effect of the light source position on the estimated slant (*F*(2, 88) = 106.53, *p* < .001) and tilt (*F*(2, 88) = 323.85, *p* < .001) of the light source on the probe. The posthoc test revealed that the three estimated slants were significantly different from each other and so were the three estimated tilts. We also found a significant main effect of diffuseness level of the scene on the estimated diffuseness on the probe (*F*(1, 44) = 6.69, *p* = .013); the estimated diffuseness level for D1 (M = 30%, *SD* = 0.011) was smaller than the estimated diffuseness level for D2 (M = 32%, *SD* = 0.008). Also, we found a significant main effect of intensity levels on the estimated light intensity on the probe (*F*(1, 44) = 603.60, *p* < .001), which was consistent with the observation in Figure 11.

Surprisingly, we found a significant main effect of the light source position on the fitted intensity on the probe (*F*(2, 88) = 28.136, *p* < .001). The posthoc test indicated that the adjusted intensity for P5 was significantly higher than for P1 (*p* < .001) and P9 (*p* < .001).

#### Part 2

**Figure 12.**
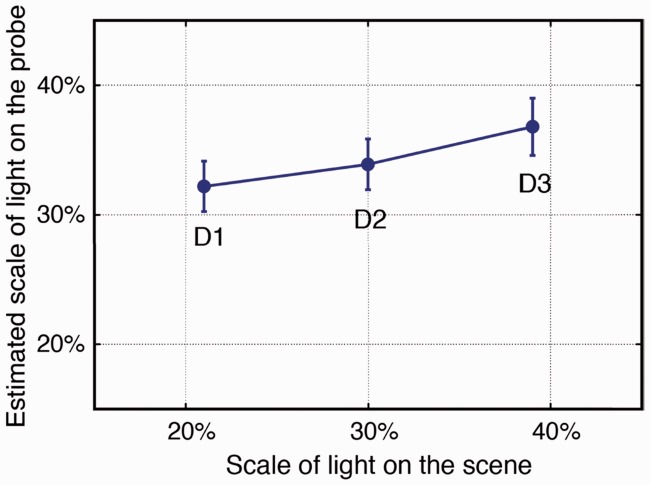
Mean-estimated scale of light on the probe as a function of the scale of light on the scene averaged over intensity levels I4 and I1. The error bars show the 95% confidence intervals (for *N* = 90 measurements).

**Figure 13.**
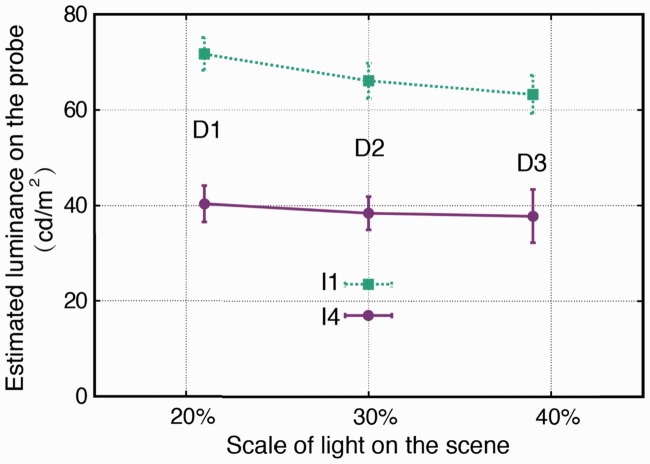
Mean-estimated light intensity (parameterized by luminance) on the probe for I4 (the lower solid line) and I1 (the upper dashed line) on the scene for diffuseness levels D1, D2, and D3. The error bars show the 95% confidence interval (for *N* = 45 measurements).

In this part, only the estimated lighting properties for P5 were investigated. A 3 (diffuseness) × 2 (intensity) repeated measures ANOVA was performed for the fitted slant, tilt, diffuseness, and intensity. As expected, we found a significant main effect of diffuseness on the fitted diffuseness (*F*(2, 88) = 6.378, *p* = .003). As Figure 12 shows, the estimated diffuseness values on the probe increased as the diffuseness on the scene increased. The posthoc test showed that the estimated diffuseness (parameterized by the scale of light) for D3 was significantly larger than that of D1 (*p* = .004) and D2 (*p* = .013). We also found a significant main effect of the intensity on the estimated light intensity on the probe (*F*(1, 44) = 335.96, *p* < .001), in that the estimated light luminance for I4 was significantly lower than that for I1, as shown in Figure 13.

Surprisingly, the analysis showed that the diffuseness levels on the scene significantly influenced the estimated intensity on the probe (*F*(2, 88) = 4.753, *p* = .011). As illustrated in Figure 13, generally, the estimated intensity on the probe decreased with the diffuseness on the scene increased. The posthoc test showed that the estimated intensity for D1 was significantly higher than for D3 (*p* = .011).

## Comparison Between Independent and Simultaneous Adjustments in Experiments 1 and 2

We analyzed the results for the lighting stimuli that were used in both Experiments 1 and 2 (marked in red in Table 7) to compare the results of independent and simultaneous estimation.

### Comparison Between Independent and Simultaneous Adjusted Light Intensity

For the light intensity, the common stimuli on the scene for Experiments 1 and 2 were I1 and I4 in position P5 with diffuseness D1. The results of paired samples *t* tests showed that the estimated intensity in Experiment 2 was higher than that in Experiment 1 both for I1 (difference: 4.9 cd/m^2^, *t*(44) = 2.21, *p* = .032) and I4 (difference: 8.3 cd/m^2^, *t*(44) = 4.11, *p* < .001), as shown in Table 8. However, we found that in Experiment 2, the simultaneously estimated diffuseness for D1 was significantly larger than in Experiment 1, both for a light intensity of I1 (difference: 11%, *t*(44) = 7.94, *p* < .001) and I4 (difference: 11%, *t*(44) = 8.58, *p* < .001). It shows the interaction between the estimated light intensity and diffuseness. When the light intensity on the probe was estimated higher than in Experiment 1, the diffuseness was estimated larger.

**Table 8.** Comparison Between Mean-Estimated Light Luminance in Experiment 1 (Inferred Independently) and in Experiment 2 (Inferred Simultaneously) for Stimuli With a Light Source in P5 Having a Diffuseness Level D1 and an Intensity of I4 or I1 on the Scene.

	Luminance (cd/m^2^)		Scale of light (%)	
Intensity levels	I1	I4	I1	I4
Scene	75.0	36.7	21	21
Probe in Experiment 1	66.8	32.1	21	21
Probe in Experiment 2	71.7	40.4	32	32

*Note.* The simultaneously estimated light diffuseness in Experiment 2 was also compared with that in Experiment 1.

### Comparison Between Independent and Simultaneous Adjusted Light Direction

For the slant and tilt, we compared the results of the stimuli for positions P1, P5, and P9 with diffuseness D1 and intensity I1 (see Table 9) in Experiments 1 and 2. The results of paired samples *t* tests indicated that neither the estimated slants nor the estimated tilts in Experiment 2 were statistically significantly different from those in Experiment 1.

**Table 9.** Comparison Between Slant and Tilt on the Probe in Experiment 1 (Inferred Independently) and in Experiment 2 (Inferred Simultaneously) for Stimuli With a Light Source in Positions P1, P5, and P9 Having a Diffuseness Level D1 and Intensity I1 on the Scene.

	Slant			Tilt		
Position	P1	P5	P9	P1	P5	P9
Scene	74°	90°	106°	73°	90°	107°
Probe in Experiment 1	75°	85°	106°	76°	82°	103°
Probe in Experiment 2	79°	87°	102°	75°	81°	102°

### Comparison Between Independent and Simultaneous Adjusted Light Diffuseness

For the light diffuseness, we compared the levels D1, D2, and D3 on the scene for intensity I1 in position P5. The results of paired samples *t* tests showed that the estimated diffuseness on the probe in Experiment 2 was significantly smaller than that in Experiment 1 for D1 (difference: –5%, *t*(44) = –3.41, *p* = .001), D2 (difference: –8%, *t*(44) = –4.47, *p* < .001), and D3 (difference: –6%, *t*(44) = –2.73, *p* = .009), as shown in Table 10. Additionally, we found that the simultaneously estimated luminance in Experiment 2 was smaller than those of the stimuli in Experiment 1 (i.e., I1) for all three diffuseness levels D1 (difference: –3.3 cd/m^2^, *t*(44) = –1.93, *p* = .06), D2 (difference: –8.9 cd/m^2^, *t*(44) = –4.86, *p* < .001), and D3 (difference: –11.8 cd/m^2^, *t*(44) = –5.97, *p* < .001). This again shows the interaction between the estimated light intensity and diffuseness; a lower estimate of diffuseness went together with a lower estimate of the intensity.

**Table 10.** Comparison Between the Mean Diffuseness in Experiment 1 (Inferred independently) and in Experiment 2 (Inferred simultaneously) for Stimuli With a Light Source in Position P5 Having an Intensity of I1 and a Diffuseness Level of D1, D2, or D3.

	Scale of light (%)			Luminance (cd/m^2^)		
Diffuseness levels	D1 (%)	D2 (%)	D3 (%)	D1	D2	D3
Scene	21	30	39	75.0	75.0	75.0
Probe in Experiment 1	37	42	45	75.0	75.0	75.0
Probe in Experiment 2	32	34	38	71.7	66.1	63.2

*Note.* The simultaneously estimated light luminance in Experiment 2 was also compared with that in Experiment 1.

### Comparison Between Independent and Simultaneous Adjusted Probe Appearances

**Figure 14.**
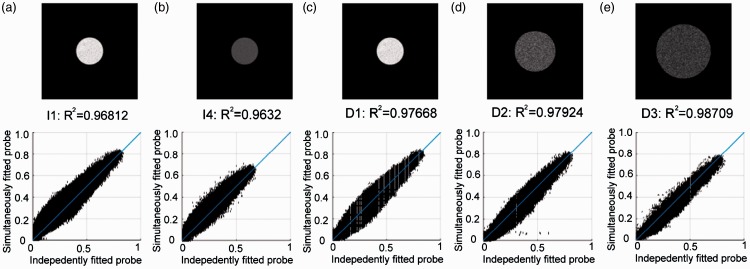
Scatterplots of the gray scale photograph of the probe taken under the average adjustment in Experiment 1 versus that in Experiment 2, for intensity levels (a) I1 and (b) I4 in position P5 with diffuseness D1, and for diffuseness level, (c) D1, (d) D2, and (e) D3 on the scene for intensity I1 in position P5.

Figure 14 shows pixel-wise correlations between the gray scale photograph of the probe taken under the average adjustment of Experiments 1 and 2, for the common stimuli of intensity and diffuseness estimates. The results show that the distribution in the scatter plots is well in line with the x-y diagonal, and so, that the average adjustment in Experiment 1 corresponds well to the average adjustment in Experiment 2 for the same stimuli. The correlations are quite high in that the generic R^2^ values for these straight pixel-wise regressions are all above 0.95, while correlations for arbitrary parameter settings are found to be much lower (for instance, the R^2^ for the photographs of the probe under P1 and P9 with diffuseness level D1 and intensity level I1 is 0.44). That the images for the adjusted lighting correlate so well are surprising, because the adjustments in Experiments 1 and 2 are performed in different sessions, and the probe appearance could not be compared directly between the sessions. Thus, consistent with the previous finding, it indicates that the observers have an internal expectation of how a probe would look like and this expectation is rather consistent. Furthermore, the observers are able to rely on this expectation to adjust the lighting properties.

## Discussion

### Methods

In this study, we investigated observer’s abilities to infer light field properties in a real scene both independently and simultaneously using an adjustment method and optical mixing of a scene and rough probe. In a previous study by Xia et al. (2014b), the same scene was used but with a forced-choice method, in which observers indicated yes or no to “visual fit.” The results of the previous study showed that the observers had difficulty to see the mismatch between the probe and the scene for scales of light of 24% and 43%. However, in this study, their estimated diffuseness for a scale of light of 21% was statistically different from that of 39%. Similarly, in the previous study, the observers could not see the mismatch between I1 and I3 and between I3 and I5 when the probe was illuminated by the lower intensity and the scene by the higher intensity. However, in this study, the estimated five intensity levels were all statistically significantly different from each other. We conjecture that the method of adjustment is a more sensitive method than the yes or no method. A possible reason may be that the dynamic changes in object appearance due to the adjustment can help the observers to infer the lighting conditions in our experimental setup.

### Estimated Light Intensity

We found that the observers were quite good at inferring the light intensity. Especially in Experiment 1, the independently estimated luminance values were surprisingly close to the veridical ones. It was found that in Experiment 1, the regression slope varied from 0.58 to 1.27 among 15 observers with an average value of 0.95. In Experiment 2, the slope varied from 0.48 to 1 among 15 observers with an average value of 0.78. In a previous study using photographs (Koenderink, Pont, et al., 2007), the estimated intensities were found to be monotonically related to the physical intensity values and to obey the “inverse squares law” of photometry at least semiquantitatively. They found that the slopes of the linear regression of the intensity observations against the veridical values varied from 0.35 to 0.85 among eight observers, and the average slope was around 0.58. Photographs are different from real scenes in that photographs usually have only two or three orders of dynamic range of luminance, while the real world has about 10 orders (Myszkowski, Mantiuk, & Krawczyk, 2008). The results in this study suggest that the observers might be much more sensitive to variations in light intensity in real scenes than what is reflected in photographs. However, the measurements by Koenderink et al. were done in various positions in a scene, and therefore the difference in the results may also reflect effects due to the global structure of the light field.

It has been extensively studied whether observers can extract the reflectance of surfaces under varying illumination conditions (Adelson, 2000; Gilchrist et al., 1999; Rutherford & Brainard, 2002; Todd et al., 2004). Their results showed that often the visual system can discount the illumination conditions and viewing conditions resulting in lightness constancy. Human’s lightness perception is not perfectly veridical but without the ability of inferring the luminous flux falling on a surface, observers would be unable to infer surface reflectance. Our results for the intensity adjustments can be seen as a direct proof of this ability.

### Estimated Light Direction

For the light direction, the independently estimated directions and the simultaneously estimated ones showed no statistically significant difference, and the observers could fit the light direction rather well in our real scene. The average fitted slant and tilt were overall within a few degrees of the veridical values. However, both in Experiments 1 and 2, we found that the standard deviation for the inferred tilt was smaller than for the inferred slant. This result is consistent with existing literature (Koenderink, Pont, et al., 2007; Koenderink et al., 2003; Pont & Koenderink, 2007). Pont et al. introduced the concept of “surface illuminance flow” to describe the 3D texture gradients due to illuminated surface mesorelief (Koenderink, Van Doorn, & Pont, 2007; Pont & Koenderink, 2004). The variation of illuminance on the macroscale is usually denoted as “shading.” The “surface illuminance flow” provides cues about the lighting additional to the “shading.” Xia et al. (2014b) showed that the “surface illuminance flow” over a rough sphere was indeed helpful in estimating the light direction and diffuseness in real scenes. Besides the “illuminance flow” over rough surfaces, Pont and Koenderink (2004) showed that the “illuminance flow” could also be estimated from images of arbitrary natural images. The shading patterns and patterns of shadow contour variations over the scene give information about the “illuminance flow” over the scene and thus provide salient information about the illumination direction. However, it is difficult to estimate the depth of the light source (“slant angle”) due to basic ambiguities in the (retinal) images, such as the bas-relief ambiguity (Belhumeur, Kriegman, & Yuille, 1999). The results of the current study suggest that the “illuminance flow” over the scene was also used as a cue for the illumination.

### Estimated Light Diffuseness

In our study, we found that the observers could infer the light diffuseness, but, consistent with previous research (Koenderink, Pont, et al., 2007; Pont & Koenderink, 2007), in a very coarse manner. The sequence of adjustment of the three lighting properties in Experiment 2 was recorded. We checked all the 630 stimuli (15 participants × 14 stimuli × 3 times) and found that the light direction was chosen as the first property to adjust for 290 times, and the intensity for 310 times, while the diffuseness only for 30 times. This result suggests that the diffuseness is the most difficult property to adjust. The difficulty in inferring the light diffuseness was also reflected in the observers’ performance.

Our results also show that the fitted diffuseness levels were generally larger than the veridical values especially for D1 (scale of light of 21%) and D2 (scale of light of 30%). According to Frandsen, scales of light of 21% and 30% represent light fields dominated by parallel light. Koenderink, Pont, et al. (2007) also found that observers inferred the light to be more diffuse than the veridical values in their collimated light condition. Furthermore, five recent psychophysical studies have investigated assumptions that observers make about light diffuseness when estimating shape and reflectance. They found that observers tend to assume high levels of diffuseness—often higher than the actual diffuseness of the light in the scene being viewed (Bloj et al., 2004; Boyaci, Doerschner, & Maloney, 2004, 2006; Boyaci et al., 2003; Schofield, Rock, & Georgeson, 2011). Morgenstern et al. (2014) made 570 measurements of the diffuseness levels in natural scenes and found that the mean diffuseness level indicator Illuminance Contrast Energy (ICE) ranged from 0.41 to 0.66 in these scenes. ICE ranges from 0 for a completely uniform, ambient light source to 1.29 for a distant point light source. So, the range 0.41 to 0.66 represented relatively high diffuseness levels. Thus, most of the light encountered in daily life is relatively diffuse instead of being collimated. This might explain the observers’ bias toward more diffuse rather than collimated light.

### Interactions Between the Estimated Light Properties

**Figure 15.**
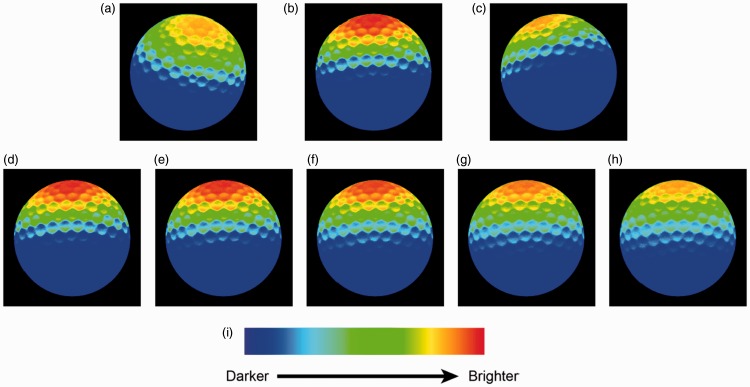
Color-mapped photographs of the probe under different light directions (the first row) or light diffuseness levels (the second row). The photographs were taken along the observers’ viewing direction. First row: The light source on Screen C is in position (a) P1, (b) P5, (c) P9; Second row: The light source located in position P5 with diffuseness levels (d) D1, (e) D2, (f) D3, (g) D4, (h) D5. (i) Legend of the color map, from left to right, blue represents the darkest area and red represents the brightest area.

We found an interaction between the estimated light intensity and light direction and an interaction between the light intensity and light diffuseness in Experiment 2. These interactions suggest that the observers are quite sensitive to the luminous flux falling on the probe. Figure 15 illustrates the variation in physical brightness of a probe under lighting stimuli in different directions (in the first row) or with different diffuseness levels (in the second row). The light source in P5, compared with that in P1 and P9, generates more luminous flux in the center of the scene where the probe was supposed to be located. As a result, the observers might have adjusted the intensity above the probe in P5 to be higher than that in P1 and P9. Similarly, with smaller diffuseness levels on the scene, more luminous flux falls on the central area of the scene. Thus, the observers might have estimated the intensity for D1 to be higher than for D3.

In a similar experiment performed on a display screen, Koenderink, Pont, et al. (2007) did pixel-wise regressions of the actual probe images to predicted images that were computed for the veridical settings and found that these correlations were quite high. In a previous study, it was found that observers judged the fit to be better if the average luminance of the visible parts of the probe was closer to the average luminance of the visible parts of the scene objects (Ling Xia et al., 2014b). Combining the findings earlier, it indicates that the observers rely on their expectation of the probe appearance (i.e., their visual light field as defined by Koenderink et al.) to judge whether the probe fits the scene, within which the brightness is an essential cue. The light intensity, direction, and diffuseness are not “orthogonal” in influencing the appearance of the probe. For instance, as the second row in Figure 15 shows, with higher diffuseness levels, the top of the probe gets darker. Comparing Experiment 1 with Experiment 2, for the stimuli whose diffuseness was inferred to be larger in Experiment 2, the intensity was inferred to be higher and vice versa (see Tables 8 and 10).

In addition to the intensity-diffuseness and the intensity-direction interactions we found here, Pont and Koenderink (2007) found that simultaneous judgments of light direction and light diffuseness from the appearance of a matte white sphere interact with each other (i.e., the “diffuseness-direction interaction”). Such perceptual interactions are to be expected on the basis of image-based analyses of the incoming optical structure or proximal stimulus, since there is no unique solution to the so-called *inverse problem* (inferring the physical light properties from the appearance of the probe object). Thus, the image (of the scene and of the probe) is ambiguous with regard to certain light properties, or, in other words, the light properties are confounded in the resulting object appearance. This applies to any natural luminous environment. Nevertheless, we argue that simultaneous adjustment of the light intensity, direction, and diffuseness is a suitable method for perception experiments and lighting interfaces, especially in combination with the use of a rough white matte spherical probe. Despite the image ambiguities, the light intensity, direction, and diffuseness have well distinguishable effects on the appearance of such objects. Indeed our results of Experiment 2 show that, using a rough golf ball, the interactions were relatively small, and the three parameters were adjusted quite accurately—in comparison to the results of Experiment 1 and former results using a yes or no fit method (Ling Xia et al., 2014b). To summarize, simultaneous adjustment of all first-order lighting properties is a feasible approach in a lighting interface, in which the designers or observers can use these properties as “buttons” to “tune the light.” More specifically, our results show that an interface on which these properties can be adjusted in a blended manner, in combination with a visual representation of the resulting appearance, is a practically feasible manner to vary the spatial distribution of the light (as an observer in a perception experiment or as a designer designing a light plan). However, we should keep in mind that perceptual interactions between these lighting properties may happen because of basic image ambiguities.

Since the light intensity is directly correlated with the brightness, it is probable that the observers attributed part of the brightness loss because of the higher estimated diffuseness to lower intensity, and they compensated for the lost luminous flux by adjusting the intensity to be higher.

## Summary

In this study, we used an adjustment method to measure the observers’ abilities of inferring the light intensity, direction, and diffuseness. Two experiments were conducted using a “probe object” approach in an experimental setup, which optically mixed a scene and a probe together. We used a matte white rough spherical probe, which provides surface illuminance flow in addition to shading cues. In Experiment 1, the observers were asked to estimate the three basic lighting properties independently, while in Experiment 2 they were asked to estimate these lighting properties simultaneously.

In Experiment 1, we found that the observers were quite sensitive to the variation of light direction and light intensity, but estimated the light diffuseness much more coarsely. In Experiment 2, we found that although the inferred light properties interacted slightly, the observers were well able to infer all three properties simultaneously. Thus, the simultaneous adjustment method using an optically mixed scene and probe was proved to be an efficient way to measure observers’ sensitivity for light field characteristics in real scenes. Simultaneous adjustment of light intensity, direction, and diffuseness of light is thus feasible in lighting interfaces, in which the users can use these properties as “tuning buttons.”
